# Nonlinear effects of climate change on outdoor activities and potential feedback pathways: a systematic review

**DOI:** 10.3389/fpubh.2026.1836657

**Published:** 2026-05-28

**Authors:** Yuqi Zhu, Junyi Guo, Jinhui Zhang, Wenhao Du, Zhiwen Guan, Hai Li

**Affiliations:** 1 School of Economics and Management, Shanghai University of Sport, Shanghai, China; 2 College of Physical Education, Hunan University, Changsha, China; 3 Department of Physical Education, Xidian University, Xi'an, China

**Keywords:** climate change, climate suitability, health risk, outdoor activities, outdoor days, systematic review

## Abstract

**Introduction:**

Climate change is reshaping the suitability, participation conditions, and risk environment of outdoor activities, but the feedback effects of outdoor activities on climate change remain less synthesized.

**Methods:**

This systematic review searched Web of Science, PubMed, EBSCO, Wiley, SpringerLink, and ProQuest for English-language studies published up to December 27, 2024. Following predefined inclusion and exclusion criteria and PRISMA 2020 reporting, 47 studies published between 2003 and 2024 were included.

**Results:**

Evidence indicates an asymmetric bidirectional relationship. Climate change affects outdoor activities through rising temperatures, extreme weather, altered precipitation and snow conditions, and environmental degradation, thereby influencing climate suitability, participation behavior, recreation demand, site availability, and health and safety risks. Five themes were identified: climate suitability and activity opportunities; participation behavior and recreation demand; health and safety risks; feedback from outdoor activities to climate change; and adaptation and mitigation strategies. Feedback evidence remains comparatively limited and mainly concerns emissions and ecological pressures associated with transportation, tourism consumption, facility operation, artificial snowmaking, energy use, and resource consumption.

**Discussion:**

Climate-change effects predominate, whereas feedback from outdoor activities plays a secondary but non-negligible role. Future research should distinguish climate suitability from actual participation and strengthen integrated assessments of carbon emissions, adaptation, risk governance, and low-carbon transition pathways in outdoor activities.

**Systematic review registration:**

https://www.crd.york.ac.uk/PROSPERO/view/CRD42025636854. Unique Identifier: CRD42025636854.

## 1 Introduction

It is well established that climate change has had profound effects across multiple sectors worldwide, including physical activity. Global warming is widely recognized as one of the most serious anthropogenic pressures on the environment. Current global warming has reached approximately 1.1 °C above pre-industrial levels, whereas the Paris Agreement aims to limit warming to 1.5–2 °C (1). Once warming exceeds certain thresholds, it may have major impacts on Earth systems, some of which are already near the lower bound of uncertain tipping-point ranges (2). If current development pathways continue without effective action, warming of 2–3 °C is likely and could produce severe consequences. Environmental changes driven by rising temperatures have made extreme climatic events increasingly common (3), and many of these changes are strongly influenced by human activities (4). Around the world, the frequency and duration of sandstorms, desertification, drought (5), cold and warm spells, wildfires and floods, and terrestrial and marine heatwaves (6) have increased (7), alongside plant diseases (8) and declines in animal biodiversity (9). The Lancet has reported that climate change poses a serious threat to human health (10), including premature mortality related to rising temperatures (11), heat illness, heat stress, and other health problems. Urgent public health responses are therefore needed. Because outdoor activities are nature-based, they are also inevitably affected by climate change.

Climate change has already affected outdoor activities. In the United States, these effects are reflected in increasing heatwaves, more frequent wildfires, reduced snowfall and snowpack, greater precipitation, and an earlier onset of spring, all of which are expected to alter nature-based outdoor activities and tourism (12). Anthropogenic climate change can influence outdoor activities in multiple ways. Studies have linked increased CO2 emissions to human outdoor activities because participation often requires additional energy consumption for transportation, regardless of travel mode. These transport-related processes increase CO2 emissions and may contribute to global warming (13). Climate warming may lengthen summer and shorten winter, increase extreme heat and drought, and reduce snowfall, thereby negatively affecting snow-based outdoor activities (14). Glacier retreat and permafrost degradation also have important implications for mountaineering, as they increase rockfall, icefall, and other hazards, making participation more dangerous and uncertain (15).

High temperatures have clear health implications for people who participate in outdoor activities and sporting events. These populations face greater heat-related health threats; for example, the annual number of heat illness cases in school sports clubs has continued to increase (16). Participants in outdoor endurance sports, such as cycling and running, face higher heat-stress risk than those engaged in short-duration sports (17). Low temperatures may also increase the risk of frostbite and other cold-related injuries during outdoor exercise. Studies have indicated that climate change can create barriers to outdoor physical activity. As temperatures rise, people may spend less time outdoors and may experience adverse health effects (18). They may also perceive that climate change reduces the health benefits or enjoyment of outdoor activities, which can lower willingness to participate in climate-affected outdoor sports (19) and ultimately affect outdoor sport participation.

Research on the relationship between climate change and outdoor activities has grown, but three limitations remain. First, existing studies have focused mainly on the effects of climate change on outdoor activities, particularly the effects of heat exposure, heat stress, extreme weather, reduced snowfall, and seasonal change on activity conditions, participation experience, and health and safety. Much less attention has been paid to the climatic feedback generated by outdoor-activity-related transportation, tourism consumption, infrastructure construction, site maintenance, and artificial snowmaking. In other words, the evidence base is directionally asymmetric: evidence that climate change affects outdoor activities is relatively strong, whereas evidence that outdoor activities feed back into climate change remains scattered. Second, outcome indicators are not fully consistent across studies. Some studies examine climate suitability or the number of suitable outdoor days, whereas others examine actual participation behavior, tourism demand, or health and safety outcomes. Climate suitability indicators can reflect the potential feasibility of outdoor activities under specific climatic conditions, but they are not equivalent to real participation frequency, participant numbers, or travel behavior. It is therefore necessary to distinguish different outcome endpoints in a review and avoid conflating climate suitability with actual outdoor activity behavior. Third, the effects of climate change on outdoor activities show marked regional heterogeneity and activity-type differences. The same degree of warming may extend the outdoor activity season in cold regions, but increase heat exposure risk and reduce safe activity time in hot regions. Explaining climate impacts only through a single climatic factor or a single activity type is therefore insufficient. A more comprehensive interpretation must consider regional baseline climate conditions, activity-related thermal thresholds, environmental exposure levels, and participant characteristics. This study introduces a threshold-based perspective (20) to explain why similar climatic changes can produce different, or even opposite, outcomes across regions and activity types.

To avoid conceptual ambiguity caused by the overlapping use of terms such as outdoor activities, outdoor sports, outdoor physical activity, and outdoor recreation, this review uses outdoor activities as the overarching object of analysis. In this article, outdoor activities refer primarily to physically engaged behaviors that occur in natural environments or open spaces, including outdoor sports, outdoor physical activity, and outdoor recreational activities. Outdoor sports emphasizes activity types with clear sport-specific attributes, exercise forms, or training purposes, such as running, cycling, snow and ice sports, water sports, and mountaineering. Outdoor physical activity places greater emphasis on routine, health-promoting bodily participation. Outdoor recreational activities emphasize leisure, experiential, and entertainment attributes in natural environments. Given differences in terminology across the literature, this review uses outdoor activities as the umbrella concept in the overall discussion, while retaining subordinate concepts such as outdoor sports when discussing specific research settings, activity types, or terminology used in original studies. This approach improves conceptual consistency and inclusiveness throughout the article.

Against this background, this review aims to systematically synthesize the relationship between climate change and outdoor activities. Specifically, it focuses on the effects of climate change on climate suitability, participation behavior, tourism demand, and health and safety outcomes in outdoor activities. It also summarizes the potential feedback of outdoor-activity-related transportation, tourism consumption, infrastructure construction, and site maintenance on greenhouse gas emissions and climate change. This review does not assume that the two directions of the relationship have equally strong evidence bases. Instead, it treats the effects of climate change on outdoor activities as the primary object of synthesis and the feedback from outdoor activities to climate change as a supplementary line of discussion. On this basis, it further analyzes how the direction and magnitude of effects differ across regions, climate backgrounds, and activity types, and proposes strategies for adapting to climate change and reducing climate impacts. The findings may inform outdoor activity management, sport tourism planning, public health interventions, and related policy development.

Specifically, this review addresses the following research questions:

1. How does climate change affect the climate suitability, actual participation behavior, tourism demand, site availability, and health and safety risks of outdoor activities?
2. Why do the effects of climate change on outdoor activities differ, and sometimes move in opposite directions, across regions, climate backgrounds, and activity types?
3. How do outdoor activities and their related transportation, tourism consumption, infrastructure operation, and resource use feed back into greenhouse gas emissions and ecological environmental pressures?
4. In response to environmental constraints and health and safety risks under climate change, how should outdoor activities pursue adaptation and mitigation at the levels of individual behavior, activity organization, infrastructure development, and policy governance?

## 2 Materials and methods

This review used a systematic review approach to identify, screen, and synthesize literature on the relationship between climate change and outdoor activities (21). To ensure transparency and reproducibility in the search, screening, and synthesis processes, this review was reported in accordance with the PRISMA 2020 guidelines and followed five steps (22, 23). The review protocol was prospectively registered in PROSPERO (CRD42025636854).

1. Define the research questions (RQs).
2. Specify the inclusion and exclusion criteria.
3. Develop the review protocol and search strategy.
4. Remove duplicates and assess eligibility.
5. Code and synthesize the included studies.

### 2.1 Step 1: define the research questions (RQs)

Based on the umbrella definition of outdoor activities used in this review and the differences in outcome indicators across the existing evidence, this study addresses the following questions:

RQ1: How does climate change affect the climate suitability, actual participation behavior, tourism demand, site availability, and health and safety risks of outdoor activities?

RQ2: Why do the effects of climate change on outdoor activities differ, and sometimes move in opposite directions, across regions, baseline climate conditions, and activity types?

RQ3: How do outdoor activities and related transportation, tourism consumption, infrastructure construction, site maintenance, and resource use feed back into greenhouse gas emissions and ecological environmental pressures?

RQ4: In response to environmental constraints and health and safety risks under climate change, how should outdoor activities pursue adaptation and mitigation at the levels of individual behavior, activity organization, infrastructure development, and policy governance?

### 2.2 Step 2: specify the inclusion and exclusion criteria

The inclusion criteria for this study covered four aspects.

First, each study had to address both climate-change-related content and outdoor-activity-related content. Climate-change-related content included, but was not limited to, climate change, global warming, greenhouse effect, rising temperature, climate adaptation, climate resilience, carbon dioxide, extreme weather, heat exposure, thermal comfort, and thermal suitability. Outdoor-activity-related content included outdoor activities, outdoor sports, outdoor recreation, adventure sports, physical activity in natural environments, and outdoor exercise behavior.

Second, this review uses outdoor activities as an umbrella concept and does not exclude studies focused on a specific outdoor activity or sport. Studies of running, cycling, skiing, skating, mountaineering, water sports, outdoor recreation, nature-based tourism, or other specific activities were included if their research questions reflected the effects of climate change on climate suitability, actual participation behavior, tourism demand, site availability, or health and safety risks, or if they reflected feedback from these activities to greenhouse gas emissions and ecological environmental pressures. By contrast, studies that discussed training, techniques, athletic performance, equipment, event management, or general tourism issues in a specific outdoor sport, but did not involve climate change, climate suitability, environmental exposure, health risk, carbon emissions, or ecological environmental impacts, were excluded.

Third, this review included research articles and review articles. Review articles were included because they help identify existing research themes, theoretical perspectives, and evidence gaps, and because they provide background reference for the synthesis. Conference papers, dissertations, books, and patents were not included, mainly because their peer-review standards, research quality, and reproducibility differ substantially.

Fourth, only English-language studies were included. Non-English studies, studies for which full text could not be obtained, non-human activity studies, indoor activity studies, and studies unrelated to the research questions of this review were excluded.

### 2.3 Step 3: develop the review protocol and search strategy

This study searched six databases: Web of Science, PubMed, EBSCO, Wiley, SpringerLink, and ProQuest. The search covered the period from database inception to December 27, 2024. To identify studies on the relationship between climate change and outdoor activities, the search used Boolean logic (24) to combine climate-change-related terms with outdoor-activity-related terms. The initial search string was: (Climate Change OR Global Warming OR Greenhouse Effect OR Rising Temperature OR Mitigation and Adaptation to Climate Change OR Uncertainty Surrounding Climate Change OR Climate Resilience OR Carbon Dioxide) AND (Outdoor Sports OR Outdoor Activities OR Physical Exercise in the Outdoors OR Outdoor Recreation OR Adventure Sports OR Team Sports in the Outdoors OR Physical Activity in Natural Environments OR Outdoor Exercise Behavior).

It should be noted that the initial search strategy centered on the two core concepts of climate change and outdoor activities. It did not explicitly include all terms related to climate suitability, such as mild weather, favorable weather, thermal suitability, thermal comfort, outdoor days, and activity-friendly climate. As a result, some studies that primarily used climate suitability, outdoor days, or thermal comfort as their main terminology, but did not directly use climate change or outdoor activities in the title, abstract, or keywords, may not have been fully captured.

To reduce the impact of this issue on the completeness of the review, studies related to climate suitability for outdoor activities were further identified during title, abstract, and full-text screening. Specifically, studies were included in the full-text assessment if their research questions, variables, or outcome indicators involved outdoor days, thermal suitability, thermal comfort, favorable weather conditions, activity-friendly weather, or climate suitability, and if they could answer how climate change affects outdoor activity suitability, participation conditions, tourism demand, or health risks. At the same time, such studies were classified separately under climate suitability in the results synthesis and were not treated as direct evidence of actual outdoor activity participation behavior.

Nevertheless, the fact that the initial search string did not explicitly include all of the climate-suitability terms above remains one limitation of this study. Accordingly, the Discussion section acknowledges that future reviews could more systematically incorporate terms such as mild weather, thermal suitability, thermal comfort, outdoor days, and climate suitability into search strategies to improve coverage and reproducibility in studies of climate suitability.

### 2.4 Step 4: remove duplicates and assess eligibility

Deduplication and screening were conducted in two stages. In the first stage, duplicate records were removed using the automatic deduplication function in EndNote 8.0. Because duplicate records may remain after automatic deduplication due to inconsistencies in titles, journals, publication years, or database record formats, the second stage involved manual deduplication. After all records were imported into EndNote, the researchers sorted them successively by Year, Title, and Journal and conducted three rounds of manual checking to minimize missed duplicates.

After deduplication, studies were first screened by publication type, and non-research outputs such as books, conference papers, patents, and dissertations were excluded. Titles and abstracts were then screened to exclude records clearly unrelated to the research topic. Finally, the full texts of the remaining studies were assessed against the inclusion and exclusion criteria to determine whether they fell within the scope of this review.

During screening, this review specifically distinguished studies of specific outdoor activities from single-sport studies unrelated to the topic of this article. Studies focused on a single outdoor activity or sport were not automatically excluded. If such studies addressed climate change, climate suitability, extreme weather, heat exposure, snowfall change, natural environmental change, participation behavior, tourism demand, health and safety risks, or environmental feedback, they were included in the analysis. Only studies that involved a specific outdoor sport but whose core content was unrelated to climate change or environmental impacts were excluded (Table 1).

**Table 1:** Inclusion and exclusion criteria.

**Category**	**Inclusion criteria**	**Exclusion criteria**
Topic relevance	Studies addressing both climate change-related issues and outdoor activity-related issues	Studies unrelated to either climate change or outdoor activities
Climate-related concepts	Climate change, global warming, rising temperature, extreme weather, climate adaptation, climate resilience, carbon emissions, heat exposure, thermal comfort, thermal suitability, climate suitability	Studies discussing general weather conditions without any link to climate change, climate suitability, environmental exposure, or carbon emissions
Outdoor activity-related concepts	Outdoor activities, outdoor sports, outdoor recreation, adventure sports, outdoor physical activity, physical activity in natural environments, outdoor exercise behavior	Indoor-only activities, non-human activities, or activities unrelated to outdoor physical, recreational, or sport participation
Specific activity studies	Studies on a specific outdoor activity, such as running, cycling, skiing, skating, mountaineering, hiking, water sports or outdoor tourism, were included if they addressed climate change, climate suitability, participation, tourism demand, health risk, safety risk, carbon emissions or environmental impacts	Studies on a single sport or activity were excluded only when they focused solely on training, technique, performance, equipment, event management, or general tourism without relevance to climate change or environmental impacts
Article type	Research articles and reviews	Academic dissertations, conference papers, books, patents, editorials, commentaries, and non-peer-reviewed materials
Language	Written in English	Written in languages other than English
Accessibility	Full text available	No full text available

### 2.5 Step 5: code and synthesize the included studies

For the final set of included studies, this review first extracted basic information, including author, publication year, study area, study type, activity type, research method, and main conclusion. The studies were then coded into the following categories according to the research questions and the endpoint distinctions emphasized in the review comments: 1. climate suitability studies, including studies that assess the suitability of outdoor activities based on climatic conditions, such as outdoor days, thermal suitability, thermal comfort, climate suitability, and activity-friendly weather; 2. actual participation behavior studies, including studies of outdoor activity frequency, participation time, activity intensity, travel behavior, and event or activity attendance; 3. tourism demand and site availability studies, including studies of ski tourism, nature-based tourism, outdoor recreation destination choice, site opening conditions, snow conditions, and seasonal change; 4. health and safety risk studies, including studies of heat stress, heat illness, cold injury, air pollution exposure, extreme weather exposure, injury risk, and perceived psychological risk; and 5. climate feedback and environmental impact studies, including studies of transportation, tourism consumption, infrastructure construction and operation, artificial snowmaking, energy use, water resource consumption, and greenhouse gas emissions. In addition, the CASP tool was used to appraise the quality of the included studies in terms of research question, methodological design, data sources, interpretation of findings, and study limitations.

## 3 Results

### 3.1 Included studies

This review implemented the five-step screening procedure. A total of 9,834 records were identified through six databases (Web of Science, PubMed, EBSCO, Wiley, SpringerLink, and ProQuest) and manual searching. Of these, 4,604 records were retrieved from Web of Science, 788 from PubMed, 685 from EBSCO, 1,072 from Wiley, 2,159 from SpringerLink, 505 from ProQuest, and 21 from supplementary manual searching.

**Figure 1:**
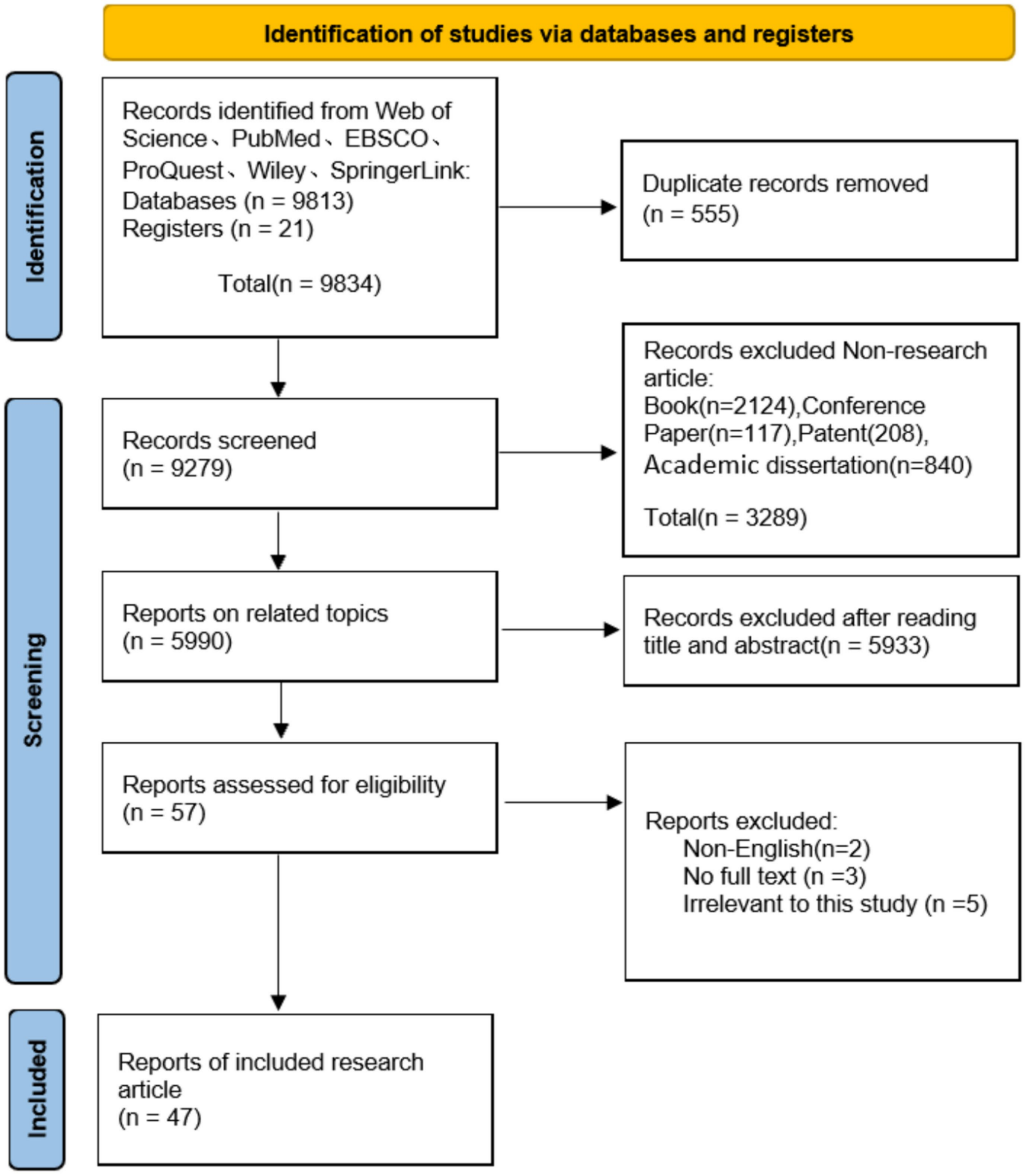
Literature screening flowchart.

After automatic deduplication in EndNote and manual verification, 555 duplicate records were removed. Books, conference papers, patents, dissertations, and other non-research publication types were then excluded, leaving 5,990 records for title and abstract screening. Junyi Guo and Yuqi Zhu, both with relevant academic training and review experience, independently screened titles and abstracts. A total of 5,933 records unrelated to the research topic were excluded, and 57 studies entered full-text assessment. Finally, 10 studies were excluded because they were non-English, full text was unavailable, or the topic was not relevant to this review. A total of 47 studies were included. The included studies were published between 2003 and 2024 and comprised 11 review articles and 36 empirical research articles. The CASP quality appraisal showed that 74% of the studies were rated High and 26% were rated Moderate. The literature screening process is shown in Figure 1, and the basic characteristics of the included studies are presented in Table 2.

**Table 2:** Characteristics of included studies.

**Representative studies**	**References**	**Title**	**Study aim**	**Study area/sample/data source**	**Article form**	**Research method**	**Main outcome**	**Thematic category**	**Quality**
1	Townsend et al. (18)	Too hot to trot? Exploring potential links between climate change, physical activity and health	To examine potential links between climate change, physical activity, and health.	Published literature on climate change, physical activity, and health.	Review	Literature review	Climate change may reduce outdoor physical activity, especially in already hot regions, with adverse health implications.	Physical activity and health risk	High
2	Shaw and Loomis (25)	Frameworks for analyzing the economic effects of climate change on outdoor recreation	To analyze how climate change may affect outdoor recreation from an economic perspective.	Outdoor recreation and economic framework literature.	Review	Economic framework analysis	Warming may increase some warm-weather recreation activities but reduce snow-dependent activities such as skiing.	Recreation demand and economic impact	Moderate
3	Bafaluy et al. (42)	Present and future climate resources for various types of tourism in the Bay of Palma, Spain	To assess present and future climate suitability for different tourism and outdoor recreation activities.	Bay of Palma, Spain; tourism climate data.	Journal article	Climate Index for Tourism assessment	Football remains highly suitable; marine sports potential increases; hiking suitability declines; seasonal suitability shifts toward spring and autumn.	Tourism climate resources	High
4	Brocherie et al. (48)	Emerging environmental and weather challenges in outdoor sports	To summarize emerging environmental and weather-related risks in outdoor sports.	Published literature on outdoor sports and environmental hazards.	Review	Literature review	Heat, cold, UV radiation, lightning, strong wind, and avalanches increase outdoor sport health risks; safety guidance is needed.	Environmental and weather risk	High
5	Scott et al. (31)	The future of the Olympic Winter Games in an era of climate change	To assess future climate suitability of Winter Olympic host locations.	Former Winter Olympic host cities and climate scenarios.	Journal article	Climate impact assessment	The number of climate-suitable Winter Olympic host locations is projected to decline substantially by mid- and late-century.	Winter sport viability	High
6	Kakamu et al. (55)	Preventing heat illness in the anticipated hot climate of the Tokyo 2020 Summer Olympic Games	To identify heat-related health risks and prevention needs for the Tokyo 2020 Olympic Games.	Tokyo 2020 Summer Olympic context and heat-risk evidence.	Journal article	Climate impact assessment analysis	Global warming increases heat illness risk in outdoor sport events; multi-stakeholder prevention strategies are required.	Sport event heat safety	High
7	Kim et al. (40)	Impact of climate change on the preferred season for outdoor water activities	To assess how climate change may alter the preferred season for outdoor water activities.	Outdoor water activities and future temperature scenarios.	Journal article	Impact assessment study	Rising temperatures may extend the season for outdoor water activities and change demand and operations.	Seasonal activity shift	Moderate
8	Liu et al. (33)	Effects of climate change on outdoor skating in the Bei Hai Park of Beijing and related adaptive strategies	To examine climate change impacts on outdoor skating and possible adaptation strategies.	Bei Hai Park, Beijing; outdoor skating operation evidence.	Journal article	Survey research and review	Climate change delays opening and shortens the outdoor skating season; current adaptive measures have limited effectiveness.	Winter activity adaptation	Moderate
9	Beery (60)	Exploring the role of outdoor recreation to contribute to urban climate resilience	To explore how outdoor recreation can contribute to urban climate resilience.	Urban outdoor recreation sector and green infrastructure practice.	Journal article	Action research	Outdoor recreation can support climate adaptation through multifunctional green infrastructure and resilience-oriented planning.	Urban climate resilience	Moderate
10	Daniel et al. (61)	Climate change adaptation strategies and approaches for outdoor recreation	To identify adaptation strategies for outdoor recreation under climate change.	Outdoor recreation management literature and practice.	Journal article	Qualitative research	Climate change alters season length, user preferences, and infrastructure risks; six strategies and 25 approaches are proposed.	Adaptation management	High
11	Heaney et al. (43)	Climate change and physical activity: estimated impacts of ambient temperatures on bikeshare usage in New York City	To estimate how ambient temperature affects bikeshare use under climate change.	New York City bikeshare use and temperature data.	Journal article	Quantitative research and model prediction	Bikeshare use may increase by mid-century but decline if temperatures continue rising; seasonal effects are uneven.	Active transport and climate	Moderate
12	Orr and Inoue (58)	Sport versus climate: Introducing the climate vulnerability of sport organizations framework	To develop a framework for assessing climate vulnerability in sport organizations.	Conceptual evidence on sport organizations and climate risk.	Journal article	Conceptual framework study	Not all outdoor sports are equally affected, but all sport organizations should prepare for climate-related risks.	Sport organization vulnerability	High
13	Wallace et al. (51)	Physical activity and climate change: clear and present danger?	To synthesize evidence on climate change, physical activity, and health risks.	Published literature on climate, physical activity, and health.	Review	Systematic review	Rising temperatures may reduce physical activity and increase heat stress, especially among older and vulnerable populations.	Physical activity and health risk	High
14	Hodgson (56)	Negotiating the weather in recreational running: understanding strategies and response to inform public health	To understand how recreational runners respond to weather conditions.	Recreational runners and weather-response evidence.	Journal article	Mixed methods research	Changing weather affects running patterns, but runners often downplay temperature, rainfall, humidity, and air-quality risks.	Behavioral adaptation	Moderate
15	An et al. (28)	Projecting the influence of global warming on physical activity patterns: a systematic review	To review projected effects of global warming on future physical activity patterns.	Published studies on global warming and physical activity projections.	Review	Systematic review	Warming may increase activity in colder regions or seasons, but extreme heat, disasters, and pollution may reduce activity; effects are heterogeneous and threshold-dependent.	Physical activity projection	High
16	Chan and Wichman (44)	Climate change and recreation: evidence from North American cycling	To examine climate change effects on cycling demand.	North American cycling and weather-related data.	Journal article	Empirical research	Cycling demand shows an inverted-U temperature response; cyclists prefer warmer over colder conditions and may shift timing to cooler hours.	Cycling and recreation demand	High
17	Dickau et al. (34)	Projections of declining outdoor skating availability in Montreal due to global warming	To project future outdoor skating availability under global warming.	Montreal outdoor skating conditions and climate projections.	Journal article	Empirical research with predictive modelling	Outdoor skating availability is projected to decline sharply and may become unavailable under continued warming.	Winter activity viability	High
18	Fruhauf et al. (19)	Intention to engage in winter sport in climate change affected environments	To test whether climate-affected winter sport environments influence participation intention.	Participants exposed to climate-change winter sport scenarios.	Journal article	Experimental cross-sectional study	Climate-affected winter sport scenarios reduce intention to participate in recreational winter sports.	Winter sport participation intention	High
19	Steiger et al. (32)	The impact of climate change on demand of ski tourism - a simulation study based on stated preferences	To simulate the effect of climate change on ski tourism demand.	Ski tourists and stated-preference data.	Journal article	Field survey and experimental research	Lower snow reliability may cause substantial losses in ski tourism demand and participation.	Ski tourism demand	High
20	Proebstl-Haider et al. (41)	Climate change: Impacts on outdoor activities in the summer and shoulder seasons	To assess climate change impacts on outdoor activities in summer and shoulder seasons.	Austria; outdoor activity and climate-related data.	Journal article	Quantitative research	Rising temperature, humidity, and precipitation changes threaten hiking, sightseeing, and snow-related tourism.	Seasonal recreation impact	High
21	Steiger et al. (32)	Climate change and winter outdoor activities in Austria	To analyze climate change impacts on winter outdoor activities and the climate impact of winter tourism.	Austria; winter tourism, snow conditions, and emissions-related evidence.	Journal article	Mixed methods research	Climate change shortens snow seasons and reduces winter activity quality; winter tourism also contributes to CO₂ emissions.	Winter tourism and emissions	High
22	Ahn et al. (49)	Investigating city bike rental usage and wet-bulb globe temperature	To examine the relationship between city bike rental use and WBGT.	City bike rental records and WBGT data.	Journal article	Quantitative research	Bike use has a non-linear relationship with WBGT; heat warnings may not always reduce rental use.	Heat exposure and active transport	High
23	Dee et al. (53)	Increasing health risks during outdoor sports due to climate change in Texas: projections versus attitudes	To compare projected outdoor sport heat risks with stakeholder attitudes.	Texas; climate projections and sport practitioner attitudes.	Journal article	Interdisciplinary research	Future heat may exceed safe limits for outdoor sports, while many practitioners underestimate cancellation thresholds.	Outdoor sport heat safety	High
24	Huang et al. (47)	Analysis of the impact of urban summer high temperatures and outdoor activity duration on residents’ emotional health: Taking hostility as an example	To assess how summer heat and outdoor activity duration affect emotional health.	Urban residents and outdoor activity-duration evidence.	Journal article	Comprehensive research	High temperatures worsen negative emotions; outdoor activity below 120 min may reduce hostility, while longer exposure may increase it.	Heat and mental health	High
25	Knight and Hao (57)	Is outdoor recreation associated with greater climate change concern in the United States?	To examine whether outdoor recreation is associated with climate change concern.	United States; outdoor recreation participants and climate concern data.	Journal article	Structural equation modelling	Outdoor recreation frequency is indirectly associated with climate concern through enjoyment of nature, but not directly.	Climate perception and concern	High
26	Liu (27)	The effect of temperature on outdoor recreation activities: evidence from visits to federal recreation sites	To estimate temperature effects on outdoor recreation visits.	Federal recreation sites and temperature-related visitation data.	Journal article	Quantitative research	Temperature influences recreation visits unevenly; responses differ between warmer and colder temperature conditions.	Recreation visitation demand	High
27	Miller et al. (59)	Climate change and recreation in the Western United States: effects and opportunities for adaptation	To synthesize climate change effects and adaptation opportunities for outdoor recreation.	Western United States; outdoor recreation evidence.	Review	Integrated assessment research	Warming, drought, reduced snowpack, and wildfire reshape recreation; adaptation is needed across warm-weather, water-based, and snow-based activities.	Regional recreation adaptation	High
28	Modenese (50)	Prevention of health risks related to occupational solar ultraviolet radiation exposure in times of climate change and COVID-19 Pandemic	To review health risks from occupational solar UV exposure under climate change.	Occupational outdoor exposure literature.	Review	Review research	Climate-related warming may increase outdoor solar UV exposure and long-term health risks.	UV exposure and health risk	Moderate
29	Niedermeier et al. (46)	Intention to engage in mountain sport during the summer season in climate change affected environments	To examine intention to engage in summer mountain sport under climate-affected scenarios.	Participants in web-based experimental scenarios.	Journal article	Web-based experimental cross-sectional study	Summer mountain sport intention is less affected than winter sport intention, but emotional experience may decline.	Mountain sport participation intention	High
30	Regev and Palatnik (37)	Implications of climate change on outdoor recreation: the case of National Parks in Israel	To assess how climate-related weather variables affect national park recreation.	Israel; national park visitation and weather data.	Journal article	Econometric analysis	Temperature and rainfall negatively affect domestic and international visits; temperature is the most influential climate variable.	Nature-based tourism demand	High
31	Aslan et al. (14)	Worry about climate change of outdoor recreation participants: a case study in Turkiye	To examine climate change worry among outdoor recreation participants.	Turkiye; outdoor recreation participants.	Journal article	Quantitative research	Participants perceive risks from heat stress, snow loss, ecological change, glacier retreat, and mountain hazards.	Climate risk perception	High
32	Fan et al. (38)	Intraday adaptation to extreme temperatures in outdoor activity	To examine how people adapt the timing of outdoor activity under extreme temperatures.	Outdoor activity timing data.	Journal article	Empirical research	Extreme temperatures restrict outdoor activity and shift activity toward more comfortable times of day.	Intraday behavioural adaptation	High
33	Ferguson et al. (39)	Weather associations with physical activity, sedentary behavior and sleep patterns of Australian adults: a longitudinal study with implications for climate change	To examine weather associations with physical activity, sedentary behavior, and sleep.	Australian adults; longitudinal weather and behavior data.	Journal article	Prospective cohort study	Extreme weather reduces activity and sleep; physical activity shows an inverted-U relationship with maximum temperature.	Weather and health behavior	High
34	Grofelnik et al. (13)	Evaluating the travel carbon footprint of outdoor sports tourists	To evaluate the travel carbon footprint of outdoor sports tourists.	Outdoor sports tourists and travel-related carbon data.	Journal article	Empirical research	Transport and activity-related energy use generate CO₂ emissions; income is positively related to emissions, while education is negatively related.	Carbon footprint and mitigation	High
35	Hsu and Sharma (45)	Disaster and risk management in outdoor recreation and tourism in the context of climate change	To synthesize disaster and risk management issues in outdoor recreation and tourism under climate change.	Literature on outdoor recreation, tourism, climate hazards, and risk management.	Review	Systematic review research	Climate change increases risks to experience value, landscape quality, biodiversity, comfort, and safety; big data and sensor-based tools can support risk reduction.	Disaster and risk management	High
36	Malinović-Milićević et al. (30)	Evaluation of outdoor thermal comfort conditions: evidence from the Serbian major ski resort over the last 30 years	To evaluate long-term changes in outdoor thermal comfort at a ski resort.	Serbian major ski resort; 30-year climate comfort data.	Journal article	Empirical research	Warm or thermally favourable days increase, while extreme and strong cold-stress days decrease; adaptation strategies are needed.	Thermal comfort and ski tourism	Moderate
37	Salim et al. (15)	Climbing the Alps in a warming world: Perspective of climate change impacts on high mountain areas influences alpinists’ behavioral adaptations	To examine how perceived climate change impacts affect alpinists’ behavioral adaptation.	Alps; alpinists and high-mountain climate impact evidence.	Journal article	Empirical research	Glacier retreat and permafrost warming increase route difficulty and danger; concern about climate change promotes behavioral adaptation.	Mountain sport adaptation	Moderate
38	Sambrook et al. (54)	Outdoor sport in extreme heat: capturing the personal experiences of elite athletes	To capture elite athletes’ experiences of outdoor sport in extreme heat.	Elite outdoor athletes.	Journal article	Qualitative research	Extreme heat increasingly threatens athlete health and performance; exertional heat illness is a growing concern.	Athlete heat experience	High
39	Willwerth et al. (36)	The effects of climate change on outdoor recreation participation in the United States: Projections for the 21st century	To project how climate change may affect outdoor recreation participation in the United States.	United States; outdoor recreation participation and climate projections.	Journal article	Quantitative research	Climate change is projected to alter outdoor recreation participation across activities, regions, and seasons.	Recreation participation projection	High
40	Choi et al. (29)	Climate change impact on “Outdoor Days” over the United States	To assess how climate change may alter the availability of suitable outdoor days.	United States; outdoor-days metric and climate projections.	Journal article	Environmental health impact assessment	Climate change may change the number and spatial distribution of days suitable for outdoor activity.	Outdoor opportunity and climate suitability	Moderate
41	Franco Silva et al. (26)	Understanding the relationships between physical activity and climate change: an umbrella review	To synthesize review-level evidence on the relationship between physical activity and climate change.	Existing reviews on physical activity and climate change.	Review	Systematic review research	Climate change and physical activity interact bidirectionally: heat and pollution may reduce activity, while active lifestyles may reduce emissions.	Bidirectional activity–climate relationship	High
42	Knowles et al. (62)	Athlete insights on climate change and winter sport: impacts, thresholds, adaptations, and implications for the Future	To explore athlete perspectives on climate change impacts, thresholds, and adaptations in winter sport.	Winter sport athletes and climate-impact evidence.	Journal article	Mixed methods research	Climate change affects winter sport conditions, participation thresholds, and adaptation needs.	Winter sport athlete adaptation	High
43	Linsenmeier (35)	Global variation in the preferred temperature for recreational outdoor activity	To estimate global variation in preferred temperature for recreational outdoor activity.	Global recreational outdoor activity and temperature data.	Journal article	Econometric research	Preferred temperatures for outdoor recreation vary globally, shaping heterogeneous responses to warming.	Global recreation climate preference	High
44	Mason et al. (44)	The impact of extreme heat on mass-gathering sporting events: Implications for Australia and other countries	To review the impact of extreme heat on mass-gathering sporting events.	Literature on mass-gathering sport events and heat-related health outcomes.	Review	Systematic review research	Extreme heat increases heat stress and illness risks, especially in endurance events such as running and cycling.	Mass event heat risk	High
45	Oyama et al. (16)	Proposing adjustments to heat safety thresholds for junior high and high school sports clubs in Japan	To propose adjusted heat safety thresholds for school sports clubs.	Japan; junior high and high school sports clubs.	Journal article	Case-crossover study	Adjusted heat safety thresholds are needed to better protect school sports participants.	School sport heat safety	Moderate
46	Raines and Fitchett (52)	Exploring the risk of heat stress in high school pre-season sports training, Johannesburg, South Africa	To explore heat-stress risk during high-school pre-season sports training.	Johannesburg, South Africa; high school pre-season sports training.	Journal article	Observational study	Pre-season school sports training may expose participants to meaningful heat-stress risk.	School sport heat risk	High
47	Wilkins and Horne (12)	Effects and perceptions of weather, climate, and climate change on outdoor recreation and nature-based tourism in the United States: A systematic review	To synthesize effects and perceptions of weather, climate, and climate change on outdoor recreation and nature-based tourism in the United States.	United States; literature on outdoor recreation and nature-based tourism.	Review	Systematic review research	Heatwaves, wildfire, reduced snowpack, precipitation variability, and earlier runoff are reshaping outdoor recreation and nature-based tourism.	Weather, climate, and nature-based tourism	Moderate

### 3.2 Thematic synthesis of included studies

To improve the readability of the Results section, the 47 included studies were thematically coded according to research question, outcome endpoint, and substantive theme. The synthesis shows that existing studies are concentrated in five thematic areas: climate suitability and outdoor activity opportunities, participation behavior and activity demand, health and safety risks, feedback from outdoor activities to climate change, and adaptation and mitigation strategies. It is important to note that different themes use different outcome indicators. Outdoor days, thermal suitability, and thermal comfort mainly reflect climate suitability. Activity frequency, travel frequency, and participation intention reflect actual behavior or behavioral tendency. Tourism visits, ski-season length, and site availability reflect tourism demand and site availability. Heat stress, heat illness, and disaster exposure reflect health and safety outcomes. The thematic synthesis is presented in Table 3.

**Table 3:** Thematic synthesis of included studies.

**Theme**	**Main endpoint**	**Representative studies**	**Main findings**
Climate suitability and outdoor activity opportunities	Outdoor days, thermal comfort, climate suitability, activity-friendly weather	No. 3, 5, 7, 11, 17, 36, 40, 43	Climate change may alter the number, timing, and spatial distribution of climate-suitable days for outdoor activities. These indicators reflect potential climate suitability rather than direct participation.
Participation, recreation demand, and activity structure	Participation, visits, demand, seasonality, activity preference	No. 2, 16, 18, 19, 20, 22, 26, 30, 32, 39	Warming may increase some warm-weather or shoulder-season activities but reduce snow-dependent activities. Participation responses are heterogeneous and often non-linear.
Health and safety risks	Heat stress, heat illness, UV exposure, air pollution, injury risk, extreme weather exposure	No. 1, 4, 6, 13, 23, 24, 28, 33, 38, 44, 45, 46	Rising temperatures and extreme weather increase health and safety risks, especially for endurance events, youth sport, older adults, outdoor workers, and vulnerable populations.
Climate feedback from outdoor activities	Carbon emissions, travel footprint, infrastructure operation, artificial snowmaking, resource use	No. 21, 25, 34, 41	Evidence on feedback from outdoor activities to climate change is more limited. Existing studies mainly focus on travel-related emissions, tourism carbon footprints, infrastructure use, and indirect climate concern.
Adaptation and mitigation strategies	Heat warning, behavior adjustment, infrastructure adaptation, green infrastructure, low-carbon travel, risk management	No. 8, 9, 10, 12, 14, 27, 35, 37, 42, 47	Adaptation strategies include activity-time adjustment, heat-risk monitoring, venue management, snowmaking or infrastructure modification, disaster-risk management, and low-carbon activity organization.

### 3.3 Summary of main findings

The synthesis indicates that the relationship between climate change and outdoor activities is not a fully balanced bidirectional interaction, but is characterized by marked asymmetry in the evidence base. In the existing literature, studies on how climate change affects outdoor activities are more numerous and have more complete chains of evidence. They mainly focus on temperature change, extreme weather, altered precipitation and snow conditions, natural environmental degradation, and health and safety risks. By contrast, studies on how outdoor activities feed back into climate change remain limited. Existing evidence is mainly drawn from specific cases involving transportation, tourism-related carbon footprints, infrastructure construction and operation, artificial snowmaking, energy consumption, and water resource use. Accordingly, the Discussion treats the effects of climate change on outdoor activities as the primary object of synthesis and analyzes feedback from outdoor activities to climate change as a supplementary pathway.

To further explain regional heterogeneity in the effects of climate change on outdoor activities, this review introduces a threshold-based interpretive framework. Eltahir and Choi argue that although global warming has a broadly consistent directional trend, its regional impacts are not uniform because historical temperature distributions differ in their relationship to the temperature thresholds or optimal ranges of specific phenomena (20). This theory suggests that the effects of warming are determined not only by the magnitude of warming, but also by whether warming moves local temperature distributions into, toward, beyond, or away from the optimal temperature range for a given phenomenon. Applied to outdoor activities, the effects of climate change can be understood as the joint outcome of baseline climate conditions, activity-related thermal thresholds, and future climate shifts. In cold or relatively cool regions, moderate warming may move conditions closer to the thermal comfort range for outdoor activities and thereby increase activity opportunities. In temperate or warm-temperate regions that are already near the upper limit of thermal comfort, further warming may push conditions beyond suitable thresholds and reduce summer suitability. In tropical, subtropical, or already hot regions, additional warming is more likely to reduce outdoor activity suitability and increase heat stress and health and safety risks.

#### 3.3.1 Climate suitability and outdoor activity opportunities

The included studies show that climate change first affects the climate suitability and potential opportunities for outdoor activities. Relevant studies mainly use indicators such as outdoor days, thermal comfort, thermal suitability, tourism climate indices, or activity-friendly weather conditions. These indicators primarily reflect whether climatic conditions are suitable for outdoor activities; they do not directly measure actual participant numbers, travel behavior, or event attendance.

Temperature change is one of the main pathways through which climate change affects outdoor activity suitability. Previous studies show that temperature directly influences people’s choices to participate in outdoor activities (25). However, this effect is not a simple linear relationship. It shows clear threshold characteristics and regional variation. Warming up to a certain point may increase outdoor activity opportunities, but once temperatures continue to rise and exceed thermal comfort or activity safety thresholds, this trend may level off or reverse (26). In some low-temperature regions, when temperatures remain below 5 °C, individual travel and outdoor activity may still be constrained even if temperatures increase (27). Overall, global warming may increase the climate suitability and opportunities for some outdoor activities in winter or in colder regions, but it may also increase activity constraints through extreme heat, extreme weather, and natural disasters, and may reduce actual participation in specific regions or population groups (28).

Studies on outdoor days further illustrate regional differences in changes in climate suitability. These studies usually do not directly measure actual outdoor activity participation. Instead, they use climate suitability indicators, such as outdoor days, to assess the potential climatic conditions for outdoor activities. Outdoor days generally refer to relatively comfortable weather days that most people may consider suitable for outdoor activities. In their theory of regional climate impacts, Eltahir and Choi use outdoor days as a representative case and define the suitable daily mean temperature range for outdoor activities as 10–25 °C. Future warming may move historically colder mid- and high-latitude regions closer to this suitable range, but may move some tropical and hotter regions away from it (20). Model projections indicate that outdoor days may change to different degrees across regions by the end of this century. Climate change will also alter the seasonal suitability of outdoor activities, reducing suitability in summer while potentially increasing suitability in other seasons (29). Similarly, with global warming, the annual number of thermally suitable days may increase while days with extreme and severe cold stress may decrease. This suggests that the climatic window for outdoor activities may shift, but it does not by itself imply that actual outdoor activity participation will necessarily increase or decrease (30).

Climate change also alters the spatial and site availability of outdoor activities. Research on the Olympic Winter Games shows that 19 previous Winter Olympic host cities had suitable climatic conditions during 1981–2010, but under a high-emissions scenario the number of climatically suitable host cities is projected to decline substantially by the 2050s and 2080s (31). Ice- and snow-based activities, such as outdoor skating and skiing, are highly dependent on natural snow conditions, ice stability, and season length. Climate change may delay opening dates, shorten operating seasons, and reduce activity availability (32–34). Outdoor recreation in the western United States is also affected by increasing heatwaves, more severe wildfires, reduced snowfall and snowpack, greater precipitation variability, and earlier spring runoff (12). In addition, glacier retreat, permafrost warming, and increasing rockfall and icefall events may affect the safety and accessibility of alpine hiking, mountaineering, and climbing (14, 15).

#### 3.3.2 Participation, recreation demand, and activity structure

Some studies directly or indirectly assess the effects of climate change on outdoor activity participation, travel, tourism demand, and activity structure. Overall, climate change may increase demand for, or the suitable season of, some warm-weather activities, water-based activities, beach recreation, and cycling. At the same time, it may reduce the available period and participation demand for skiing, outdoor skating, and other snow-based activities.

In terms of recreation and travel demand, participation in outdoor recreation often increases as weather becomes warmer, whereas participation tends to be lower at colder temperatures. One model estimates that warming of 1 °C would generate an additional 88 million trips per year (35). At the same time, people living in warmer regions tend to prefer higher temperatures for outdoor activities. In warm-climate regions, a 1 °C increase in annual mean temperature is associated with an increase of approximately 0.5 °C in the preferred daily mean temperature for outdoor activities (35). However, participation responses differ by region. For example, people in northern regions are more likely to avoid outdoor activities on the hottest days, whereas people in southern regions are more likely to continue participating under high-temperature conditions (36).

Weather variables also affect outdoor activity behavior. A series of weather variables show moderately significant associations with exercise behavior. Econometric analyses indicate that rainfall and temperature indices are negatively associated with both domestic and international outdoor activities, and that temperature has an important negative effect on domestic and international outdoor activity decisions (37). Extreme weather or temperature can constrain human physical activity and shift the timing of outdoor activity within the day (38). Under extreme weather, participants tend to be less active, sedentary time increases, and sleep duration may decrease as temperature rises (39). One study found that light physical activity was negatively associated with wind speed and positively associated with minimum temperature, cloud cover, and sunshine duration. Moderate-to-vigorous physical activity was negatively associated with minimum temperature, rainfall, and wind speed, and positively associated with sunshine duration. Maximum temperature showed an inverted U-shaped relationship with both light and moderate-to-vigorous physical activity (39).

In terms of activity structure, climate change may alter the suitability conditions, activity demand, and participation choices of different outdoor activities. For example, as temperatures rise, participation in or demand for warm-weather activities such as boating, golfing, and beach recreation is projected to increase by 14 to 36%, whereas participation in or demand for snow-based activities such as skiing may decline (25). Future temperature increases may reduce snow and ice sports, while extending the season for outdoor water activities and altering demand and operations in this sector (40). Related research also suggests that water-based activities will experience the largest increase in trips, followed by running, cycling, fishing, and hunting, whereas snow-based activities are projected to experience the largest decline in trips, followed by hiking (36). Rising temperatures and changes in relative humidity and precipitation may also threaten hiking, sightseeing, and snow-and-ice tourism in Austria (41). Another study projected that maritime sports in the bay area may increase, football suitability may remain high, jet-ski potential may improve, golf may change little, and hiking-type outdoor activities may deteriorate. Outdoor sports may increase in spring and autumn but decline in summer (42).

Cycling is a typical nonlinear case of climate change effects on outdoor activity participation. Research shows that as temperatures rise, bicycle ridership in New York City may increase by the middle of this century; however, as temperatures continue to rise, this trend may reverse, with projected increases in winter, autumn, and spring being larger than projected declines in summer (43). Cycling demand follows an inverted U-shaped relationship. Cyclists prefer higher temperatures relative to low temperatures, but demand may decline or activity timing may shift under hot conditions (43, 44). Climate change may also affect willingness to participate and emotional experience. Winter sport scenarios affected by climate change reduce participants’ willingness to engage in recreational winter sports (19). Climate also influences outdoor activity comfort, enjoyment, and the quality of recreational experience (14). Sea-level rise, beach erosion, storm surges, and coastal flooding may reduce visitor experience and willingness to return (45). Willingness to engage in summer mountain sports may be less affected, but affective valence may decline (46). High temperatures may also cause stress and fatigue, reduce pleasure, and trigger emotional responses such as depression, anger, distress, and hostility (47).

#### 3.3.3 Health and safety risks

Health and safety risks are among the most concentrated themes in the included studies. The evidence generally indicates that extreme heat, heatwaves, air pollution, ultraviolet exposure, wildfires, strong winds, lightning, avalanches, glacier retreat, and permafrost change may increase risks during outdoor activities. Endurance sports, youth sport training, outdoor physical activity among older adults, large outdoor events, and winter alpine sports are higher-risk settings.

Climate change may become a barrier to participation in physical activity, especially in higher-temperature regions. High temperatures may adversely affect health or even cause illness, reduce perceived health benefits and enjoyment from outdoor activities, and thereby reduce participation (18). Excessive heat may lead to heat injury, while excessively low temperatures may cause hypothermia and other health threats (48). Climate change may also increase the risk of insufficient snow depth for winter sports, reduce the quality, quantity, and diversity of winter outdoor activities, and generate safety risks (32). Mountaineering is also affected by environmental change, with possible increases in route technicality, danger, and uncertainty regarding suitable summer climbing conditions (15).

Extreme heat can exacerbate human disease and limit the intensity and duration of outdoor activities (49). In outdoor sports, air pollution and extreme heat may have harmful physiological effects. High temperatures have particularly important effects on health outcomes among sport-event participants, who face risks of heat stress and related illness (17). Each increment of climate warming may intensify heatwaves in most regions and further affect athlete health and performance (26). In addition, outdoor activities may increase exposure to solar ultraviolet radiation (SUVR), inducing long-term adverse health effects (50). Increases in peak temperature and drought may also threaten participant health through heat stress and increase personal safety risks during outdoor activities (14, 45).

Climate-related health risks vary across population groups. Endurance athletes, such as runners and cyclists, face higher heat-stress risk than participants in short-duration sports (17). Rising temperatures may reduce outdoor physical activity and health and increase the physiological burden of thermoregulation. Excessive heat exposure has greater effects on older adults, especially in summer, when it may increase sedentary time and reduce the intensity and frequency of outdoor physical activity, placing vulnerable populations at higher health risk (51). Children and older adults are more susceptible to extreme weather (28). In addition, heat stress is more likely to occur on artificial surfaces than on natural grass (52).

Some studies further focus on safety thresholds for outdoor sports. Projections suggest that by the middle to late 21st century, high temperatures may exceed health and safety limits for outdoor sports activities, and 4–6 h of exposure to wet-bulb temperatures above 95 °F/35 °C may be fatal (53). However, some sport practitioners still do not fully recognize the effects of climate change on the health of students and athletes or on sport programs (53). Research in the United States shows an increase in diagnoses of exertional heat illness among young athletes, and heat illness is one of the important causes of death in youth sport, with most cases related to football (54). Thousands of heat illness cases also occur each year in Japanese school sports clubs (16). Therefore, as global warming continues, health risks associated with outdoor sporting events under high temperatures, especially heat stress and extreme heat risks, require joint action by stakeholders (55).

#### 3.3.4 Climate feedback from outdoor activities

Compared with the effects of climate change on outdoor activities, fewer studies examine the reverse feedback from outdoor activities to climate change. Existing evidence is mainly concentrated in emissions related to outdoor tourism, winter sports, transportation, accommodation and catering, facility operation, artificial snowmaking, equipment use, and resource consumption. The results indicate that outdoor activities themselves do not necessarily generate substantial climate impacts directly, but their associated transportation, tourism consumption, and facility systems may produce greenhouse gas emissions and ecological environmental pressures.

Winter outdoor activities and related tourism are considered to have certain climate feedback effects. One study reported that tourism contributes approximately 8.1% of global CO2 emissions or 5.3% of CO2-equivalent emissions. A study by the Austrian Federal Environment Agency estimated that in winter tourism, 50% of CO2 emissions come from transportation, 32% from accommodation and catering, and 18% from ski-related activities (32). This indicates that in winter outdoor activity settings, carbon emissions do not mainly originate from physical activity itself, but more often from transportation, accommodation, catering, and facility services required to undertake the activity.

Previous studies have also noted that CO2 emissions are related to transportation and human activity in outdoor activities. Regardless of whether participants travel to outdoor activity destinations by car, coach, train, or airplane, travel may require additional energy consumption and produce CO2 emissions (13). Another study found that trail-running-related income was positively associated with CO2 emissions, whereas education level was negatively associated with CO2 emissions (13). These findings suggest that feedback from outdoor activities to climate change is not determined solely by the act of participating in outdoor activities. Instead, it is jointly shaped by travel distance, transport mode, consumption pattern, activity organization, and participants’ environmental awareness.

Beyond carbon emissions, some outdoor activities may generate environmental pressures through infrastructure construction, site maintenance, energy consumption, water resource use, and ecological disturbance. Winter sports and mountain outdoor activities are strongly dependent on natural environmental conditions. When climate warming reduces natural snow cover, accelerates glacier retreat, and alters permafrost, some destinations may maintain activity supply through artificial snowmaking, site maintenance, and infrastructure expansion. However, these measures may themselves increase energy use, water resource pressure, and ecological disturbance (32). At the same time, anthropogenic climate change is driving ecological change in mountain areas and increasing risks in hiking, trekking, and mountaineering through glacier melt, rockfall, and icefall events (14). Thus, mountain and winter outdoor activities are affected by climate change, while their adaptation measures may also generate new environmental costs.

It is important to distinguish these feedback pathways from broader human environmental pressures. Air pollution, urbanization, and agricultural production can affect environments for physical activity and may reduce children’s physical activity (28), but these factors cannot be directly equated with the effects of outdoor activities themselves on climate change. The reverse feedback of outdoor activities should be understood through related transportation, tourism, facility, energy, and resource-use systems, rather than by attributing all environmental degradation caused by human activities to outdoor activities.

#### 3.3.5 Adaptation and mitigation strategies

The adaptation strategies proposed in the included studies mainly include heat-risk monitoring, adjustment of activity timing, cancellation thresholds for events or training, participant education, protection of vulnerable groups, site management, green infrastructure development, disaster risk management, and facility adaptation for winter activities. Mitigation strategies mainly focus on low-carbon transportation, optimized tourism organization, reduced facility energy consumption, improved resource-use efficiency, and enhanced climate awareness among participants.

From the perspective of individual behavior and health and safety, some sport practitioners still do not recognize the effects of climate change on the health of students and athletes or on sport programs (53). Studies indicate that if no interventions are implemented, the global mean temperature may rise by 1.8 °C to 4.0 °C by 2,100, partly because of greenhouse gas emissions (51). It is therefore necessary to develop guidelines for outdoor sports under climate change (48). Among runners, some participants have insufficient awareness of weather change and often downplay factors such as temperature, rainfall, humidity, and air quality (56). Heat illness is an important cause of death among high school athletes, and its incidence may worsen in the future, requiring joint action by stakeholders (31). To protect athletes and students, accurate and timely monitoring of heat-stress risk during exercise is essential (52).

In terms of behavioral adaptation, people can adjust physical activity patterns to reduce the effects of global warming, for example by avoiding uncomfortable periods of the day, shifting outdoor activities to other times, or moving indoors when temperatures are unsuitable (28, 38). Studies also suggest that the frequency of outdoor recreation may indirectly strengthen concern about climate change through enjoyment of nature. Outdoor sports are not necessarily directly associated with climate change concern, but nature experience may shape climate awareness (57). Under appropriate transportation choices, activity organization, and everyday behavioral substitutions, physical activity may also contribute to climate change mitigation by reducing dependence on motorized transport and promoting low-carbon lifestyles, while also supporting health promotion and social support in recovery after natural disasters (26).

From the perspective of infrastructure and management, global temperature has increased by approximately 1.1 °C since 1900 (12). Demand for and operation of outdoor water activities are changing, and responses to the multiple impacts of climate change need to begin (33). Sport organizations must also prepare for climate change risks (58). For example, the outdoor skating season in Montreal has continued to shorten; if this trend continues, sites may lack usable ice by the middle of this century and adaptation measures will be needed (34). Outdoor recreation in the western United States is also changing, and related organizations and managers should adapt proactively (59). With global warming, the number of thermally suitable days may increase, while days with extreme and severe cold stress may decrease. This mainly reflects changes in the window of climate suitability and may affect the future structure of tourism and outdoor activity supply. Ski resorts therefore need to develop new strategies to adapt to a changing climate (30).

**Figure 2:**
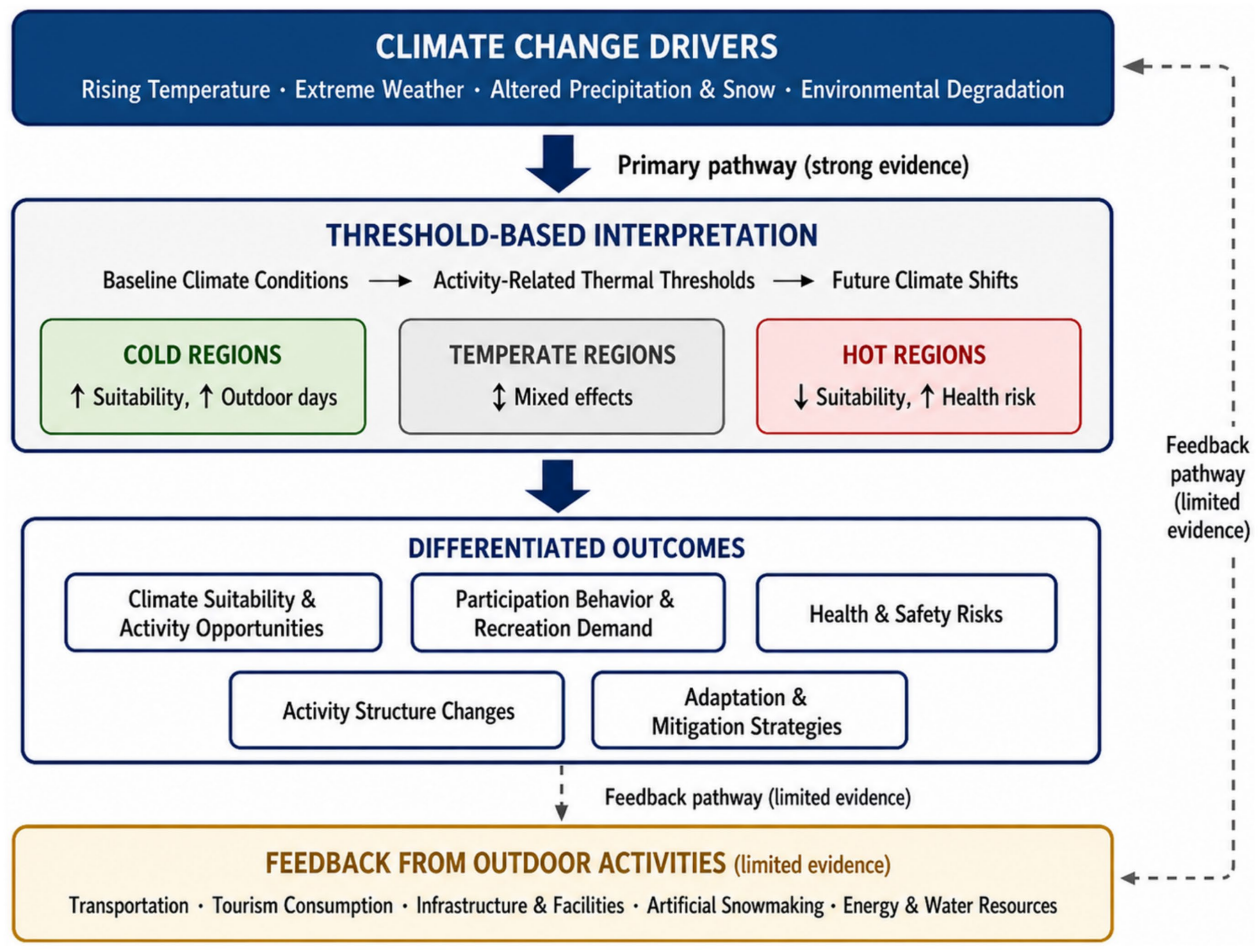
Threshold-based asymmetric framework linking climate change and outdoor activities.

Facility-based adaptation strategies include ice making, artificial snowmaking, green infrastructure, and disaster risk management. Outdoor skating operators may adapt to climate change through measures such as water spraying, and the ski industry may maintain supply through artificial snowmaking, although these measures face cost and resource constraints (33). Professional outdoor recreation sectors have identified the construction and promotion of multifunctional green infrastructure as an important direction for strengthening the role of outdoor recreation in climate adaptation (60). Big data modeling, drones, sensors, and robotic data can also be used for disaster management and risk reduction, helping tourists and emergency responders reduce damage and losses caused by climate-related disasters (45). Natural resource managers can further enhance the adaptive capacity of outdoor activity systems by protecting and maintaining critical infrastructure, strengthening the ability of natural systems to adapt to variable precipitation, managing visitation and use trends, preventing harm from extreme heat, using appropriate vegetation to improve recreational environmental resilience, and adjusting infrastructure to better capture and use natural and artificial snow (61) (Figure 2).

## 4 Discussion

### 4.1 Main findings

Based on 47 included studies, this review systematically synthesized the relationship between climate change and outdoor activities. The findings show that the relationship is bidirectional to some extent, but the evidence base is asymmetric. Existing studies focus more on the effects of climate change on climate suitability, actual participation behavior, tourism demand, site availability, and health and safety risks in outdoor activities. By contrast, evidence on the feedback from outdoor activities to climate change is relatively limited and mainly involves pathways related to transportation, tourism consumption, facility operation, artificial snowmaking, energy use, and resource use. Accordingly, this review treats the effects of climate change on outdoor activities as the primary direction of evidence and understands the feedback from outdoor activities to climate change as a supplementary pathway.

### 4.2 Threshold-based interpretation of regional heterogeneity

The effects of climate change on outdoor activities are not a simple linear process. They are threshold-based, regionally heterogeneous, and dependent on activity type. To explain the differentiated results observed across regions and activities, this review introduces the framework of baseline climate conditions–activity-related thermal thresholds–future climate shifts. This framework suggests that the direction of warming effects depends not only on the magnitude of warming, but also on the relative position of local baseline climate conditions to the suitability thresholds of outdoor activities (20). In regions or seasons that were originally cold, warming may move climatic conditions closer to the suitable range for outdoor activities. In regions that are already near the upper limit of thermal comfort or are already hot, further warming may reduce outdoor activity suitability and increase heat-stress risk. This framework helps explain why similar warming can produce opposite results across regions, and it also helps explain why snow and ice activities, water activities, cycling, hiking, and large events respond differently to climate change.

### 4.3 Endpoint distinction and evidence interpretation

Different studies use different outcome endpoints, and these endpoints should not be treated as interchangeable. Climate suitability indicators, such as outdoor days, thermal suitability, and thermal comfort, mainly indicate whether climatic conditions are suitable for outdoor activities. Actual participation behavior indicators reflect participation frequency, duration, travel frequency, or attendance. Tourism demand and site availability indicators reflect destination visits, activity-season length, and spatial conditions. Health and safety outcomes reflect heat stress, heat illness, cold injury, air pollution exposure, and disaster risk. Therefore, changes in outdoor days or thermal suitability should not be directly equated with changes in actual participant numbers or activity frequency. They should instead be interpreted as changes in potential activity opportunities at the level of climatic conditions.

### 4.4 Implications for adaptation and mitigation

In practice, outdoor activity systems need to address both adaptation and mitigation. Adaptation strategies should focus on participation constraints and health risks caused by high temperatures, low temperatures, extreme weather, natural disasters, and changing site conditions. These strategies include adjustment of activity timing, heat-risk monitoring, protection of vulnerable groups, cancellation thresholds for events and training, site safety management, and disaster risk warning. Mitigation strategies should focus on transportation modes, tourism organization, facility operation, artificial snowmaking, energy use, and resource management related to outdoor activities, with the aim of reducing carbon emissions and ecological pressures within outdoor activity systems.

### 4.5 Limitations and future research

This study has several limitations. First, the initial search strategy centered on the two core concepts of climate change and outdoor activities and did not explicitly include all terms related to climate suitability, such as mild weather, favorable weather, thermal suitability, thermal comfort, outdoor days, activity-friendly weather, and climate suitability. As a result, some studies focused primarily on climate suitability or outdoor days may not have been fully captured. Second, the evidence base is asymmetric. Most included studies examine the effects of climate change on outdoor activities, whereas fewer studies examine the reverse feedback from outdoor activities to climate change. Third, outcome endpoints differ across studies. Climate suitability, actual participation behavior, tourism demand, and health and safety outcomes are related, but they cannot be directly equated. Fourth, suitability thresholds differ across outdoor activities, and these thresholds are also shaped by age, health status, heat adaptation capacity, infrastructure, equipment conditions, and sociocultural preferences. Future research should integrate regional climate data, activity-related thresholds, actual participation behavior data, tourism demand data, and health risk data to develop more precise climate adaptation models for outdoor activities.

## 5 Conclusion

This review shows that climate change and outdoor activities have a bidirectional relationship to some extent, but the existing evidence base is asymmetric. Overall, evidence on the effects of climate change on outdoor activities is more substantial. These effects are mainly reflected in the impacts of temperature change, extreme weather, seasonal change, altered precipitation and snow conditions, and increasing natural disasters on climate suitability, participation behavior, activity structure, site availability, and health and safety risks in outdoor activities. By contrast, evidence on feedback from outdoor activities to climate change is relatively limited and is mainly concentrated in greenhouse gas emissions and ecological environmental pressures generated through transportation, tourism consumption, infrastructure construction and operation, artificial snowmaking, equipment use, and resource consumption.

Furthermore, the effects of climate change on outdoor activities are not linear. They are threshold-based, regionally heterogeneous, and dependent on activity type. This review introduces the framework of baseline climate conditions–activity-related thermal thresholds–future climate shifts to explain why similar magnitudes of warming may produce opposite outcomes across regions and activities. It is important to emphasize that climate suitability, actual participation behavior, tourism demand, and health outcomes are related but distinct endpoints. Outdoor days and thermal suitability indicators mainly reflect the potential support provided by climatic conditions for outdoor activities. They should not be directly equated with actual participant numbers, participation frequency, tourism travel, or event attendance.

Based on this understanding, the future development of outdoor activities needs to address both adaptation and mitigation. In terms of adaptation, stronger health-risk warning, activity-time adjustment, site safety management, and protection of vulnerable groups are needed under high temperatures, low temperatures, extreme weather, and natural disasters. Differentiated adaptation strategies should also be developed according to regional baseline climate conditions and activity-related thresholds. In terms of mitigation, transportation modes, tourism organization, facility operation, artificial snowmaking, energy use, and resource management related to outdoor activities should be optimized to reduce carbon emissions and ecological pressures within outdoor activity systems.

## Data Availability

Publicly available datasets were analyzed in this study. This data can be found here: https://www.crd.york.ac.uk/prospero/#searchadvanced.
